# Intergenic, gene terminal, and intragenic CpG islands in the human genome

**DOI:** 10.1186/1471-2164-11-48

**Published:** 2010-01-19

**Authors:** Yulia A Medvedeva, Marina V Fridman, Nina J Oparina, Dmitry B Malko, Ekaterina O Ermakova, Ivan V Kulakovskiy, Andreas Heinzel, Vsevolod J Makeev

**Affiliations:** 1 Research Institute for Genetics and Selection of Industrial Microorganisms, Genetika, 1st Dorozhny proezd, 1, Moscow, 117545, Russia; 2 Engelhardt Institute of Molecular Biology, Russian Academy of Sciences, Vavilova str., 32, Moscow, 199991, Russia; 3 Institute for Information Transmission Problems (The Kharkevich Institute), Russian Academy of Sciences, Bolshoy Karetny per. 19, Moscow, 127994, Russia; 4 Upper Austria University of Applied Sciences, Softwarepark 11, Hagenberg, 4232, Austria

## Abstract

**Background:**

Recently, it has been discovered that the human genome contains many transcription start sites for non-coding RNA. Regulatory regions related to transcription of this non-coding RNAs are poorly studied. Some of these regulatory regions may be associated with CpG islands located far from transcription start-sites of any protein coding gene. The human genome contains many such CpG islands; however, until now their properties were not systematically studied.

**Results:**

We studied CpG islands located in different regions of the human genome using methods of bioinformatics and comparative genomics. We have observed that CpG islands have a preference to overlap with exons, including exons located far from transcription start site, but usually extend well into introns. Synonymous substitution rate of CpG-containing codons becomes substantially reduced in regions where CpG islands overlap with protein-coding exons, even if they are located far downstream from transcription start site. CAGE tag analysis displayed frequent transcription start sites in all CpG islands, including those found far from transcription start sites of protein coding genes. Computational prediction and analysis of published ChIP-chip data revealed that CpG islands contain an increased number of sites recognized by Sp1 protein. CpG islands containing more CAGE tags usually also contain more Sp1 binding sites. This is especially relevant for CpG islands located in 3' gene regions. Various examples of transcription, confirmed by mRNAs or ESTs, but with no evidence of protein coding genes, were found in CAGE-enriched CpG islands located far from transcription start site of any known protein coding gene.

**Conclusions:**

CpG islands located far from transcription start sites of protein coding genes have transcription initiation activity and display Sp1 binding properties. In exons, overlapping with these islands, the synonymous substitution rate of CpG containing codons is decreased. This suggests that these CpG islands are involved in transcription initiation, possibly of some non-coding RNAs.

## Background

Most mammalian DNA is depleted with CpG dinucleotides [1] whose fraction in a mammalian genome is close to 0.2-0.25 of the value expected from presupposition of random distribution [2]. The shortage of genomic CpG dinucleotides is believed to be the consequence of frequent mutation of ^m^CpG to TpG dinucleotides [1,3-5]. Nevertheless, some mammalian genomic segments called CpG islands (CGIs) [3] possess a high G+C content, with a frequency of CpG close to the expected value. In bioinformatics, CGIs are usually defined as DNA segments that are longer than 200 bp, have above 50% G+C content, and have a CpG frequency of at least 0.6 of that expected assuming letters at each sequence position occurring independently at random with the given composition [3]. The number of CGIs varies substantially in different vertebrate species [4]. There are about 50,200 such CGIs in the human genome, of which approximately 29,000 are in repeat-masked sequences [5].

The increased number of CpG sites in CGIs is often correlated with low methylation of cytosine in CpG dinucleotides [6-9]. This effect is usually explained by postulating protection of these sites from DNA methyltransferase by abundant and commonly utilized DNA binding proteins including Sp1 [10], E2F [11], CTCF [12] and others. The Sp1 protein is particularly strongly implicated in CGI functioning. Gardiner-Garden and Frommer observed [3] that CGIs contain many "G/C boxes", composed of the sequence GGGCGG, demonstrated to act as binding sites for the Sp1 transcription factor [13]. Later, it was found that Sp1 can bind to both methylated and non-methylated variants of this binding site [14], and can protect non-methylated sites from methylation [10].

In his recent study Rozenberg et al. [15] demonstrated that binding sites of several regulatory proteins, including Sp1, contain a CpG pair and play an important role in the formation of sequences of mouse promoters which regulate the expression of housekeeping genes. This suggests that CGIs overlapping with promoters of housekeeping genes are related to their transcription initiation. According to [16] 60% of widely expressed human genes and up to 40% of tissue-specific genes are associated with CpG islands. It has been shown lately that 72% of all promoters have high CpG content, and only 28% are in the class with low CpG content [17].

CGIs located near 5' region of known genes account for only a fraction of all CGIs in the genome (about 25% for CGIs longer than 500 bp in the HOVERGEN compilation [18], and about 50% according to our estimations, see below). Although many non-5' associated CGIs overlap with repeats [18,19], many do not [18,20], but instead are frequently positioned 3' to known genes, overlapping with final transcribed exons [3,20]. Amazingly, CGIs located in these 3' regions have attracted almost no interest, even though these CGIs were mentioned in the publication that initially coined the term "CpG island" [3]. More recently, computational approaches have also identified intragenic CGIs that overlap neither TSS nor final exons [20], although function of these CGIs have not yet been assessed.

CGIs not associated with 5' region of any gene can perform important biological functions. For instance, a C-to-T substitution in CGI encompassing parts of exon 15 and intron 15 of *UBA1 *affects expression of this gene [21]. A CGI located within intron 10 of *KCNQ1 *and associated with an oppositely-oriented RNA transcript is involved in imprinting (paternal repression) of its locus [22]. Imprinting of *MAP3K12 *gene is associated with differential methylation of a CGI located in its last exon [23]. Many CpG islands are located near the 3' ends of genes associated with cancer development [24].

The main objective of this work was to study properties of CGIs located far from TSS of protein coding genes. We demonstrated that substantial selection pressure is applied to CpG pairs in CGIs independently from CGI location in the reference to gene starts locations, which implies functional importance of CpG pairs. We assumed with [15] that most of CGIs are involved in transcription initiation, thus one of our objectives was to study transcriptional activity of CGIs, particularly of CGIs located far from 5' regions of any protein coding gene. To do this we used **C**ap **A**nalysis **G**ene **E**xpression (CAGE) tags identified in the FANTOM project [25,26]. We also assessed the representation of binding motifs recognized by regulatory factor Sp1 in CGIs located in 5', 3' and internal gene regions, as well as out of any known genes. In addition, we re-analyzed the published ChIP-chip data on Sp1 binding in chromosomes 21-22 and compared Sp1 binding preferences in DNA not overlapping with CGIs as well as in CGIs located in different gene segments and out of any genes. Fraction of non-5' CGI strongly enriched with CAGE tags was studied with special care; we observed substantial overrepresentation of probably strong Sp1 binding sites in such CGIs and collected known reports of transcription starts sites of long non-coding RNAs associated with such CGIs.

## Results

### CGIs tend to overlap protein coding exons

Tendency of CGIs to overlap with exons has been observed many times at limited data sets [16,27,28]. As the first step of our study we decided to give a quantitative estimation of this tendency separately for exons and introns located in different gene regions. Exons and introns were categorized according to their location within the gene (see **Methods**). For exons and introns from each category the total length of overlap with CGIs was calculated. We used Monte Carlo simulations to assess the statistical significance of the observed total overlap length. A round of simulation was performed as follows. Exons (introns) were located as in the human genome and "CGIs" were sampled. Intervals between CGIs were sampled from the interval distribution evaluated from the genome, whereas the lengths of "CGIs" were shuffled length of genuine CGIs. The total overlap between exons (introns) and "CGIs" was calculated. Then the whole procedure was repeated with switched CGI and exon sets, i.e. the annotated CGIs and "simulated exons (introns)" were taken. For each category of gene elements such simulations were repeated 10,000 times and the observed values of exon (intron) overlapping with CGIs were normalized for the average simulated values.

**Figure 1:**
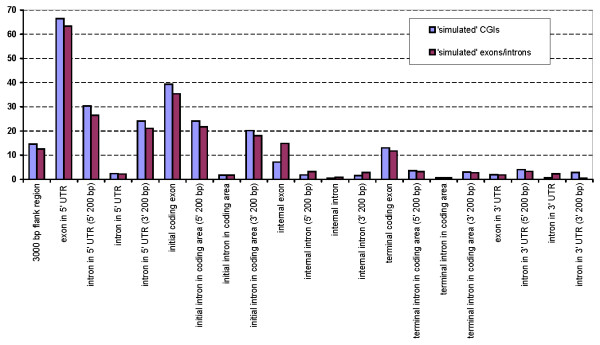
**The ratio of overlaps of bona fide CGIs and exons (introns) and overlaps of randomly positioned intervals with lengths of exon (intron) and CGI sets**. (1) Exon set is fixed, CGI set is sampled. (2) CGI set is fixed, exon set is sampled. 10,000 runs of Monte-Carlo simulation. Length distributions are computed independently for each chromosome.

Figure 1 shows that for all categories of exons (except 3' UTR exons) the fraction of their overlapping with CGIs is greater than the similar fraction for corresponding introns. Overlapping with CGIs is greatest for 5'UTRs and first coding exons. This happens because CGIs associated with promoter regions are usually longer than 1 Mb and often extended into 5' UTRs and further downstream into the coding region. Yet, the observed tendency of internal and especially of terminal exons to overlap with CGIs cannot be explained this way.

### Frequent overlapping of CGIs with exons cannot be explained as misinterpretation of GC-rich exons as CGIs

It is known that exons are usually more GC-rich than introns [29]. At the same time, the algorithm for CGI computational identification uses the increased C+G content of a test DNA segment as one of the CGI conditions. On the other hand, a CG-rich exon can have an increased number of CpG dinucleotides owing to its specific amino acid composition, e.g. many arginine codons. Therefore, this exon would be misidentified as CGI, and many such events would explain an increased overlapping between CGIs and exons.

A more interesting alternative explanation of frequent overlapping of CGIs with exons is that it is caused by the common preferences of both segments to be located in some particular DNA regions. In this case the terminal intron segments that are close to exons would also overlap with CGIs more frequently than internal segments of long introns. To test this, we selected 200 bp intron fragments adjacent to donor and acceptor splice sites. As in the previous section, we used Monte Carlo simulations to assess expectation of the observed overall overlap lengths.

Figure 1 shows the normalized intersection of CGIs with the terminal regions of introns. One can see that the normalized overall overlapping of intron terminal regions with CGIs is more similar to the values for CGI overlapping with exons than to the values for CGI overlapping with the internal segments of introns. Table 1 also shows that CGI overlapping with internal segments of introns is less likely than CGI overlapping with randomly positioned intervals of the same length. Therefore, CGIs have some tendency to avoid being buried within introns. This agrees better with the tendency that both exons and CGIs exhibit a preference to occupy the same DNA regions with yet unknown properties and CGIs overlapping with exons often extend significantly into introns.

**Table 1:** Ratio of bona fide CGIs-exons (introns) overlap and "simulated" overlap average

Gene region	Bona fide overlap length/Simulated overlap average	
	"Simulated" CGIs	"Simulated" exons
3000 bp flank region	14.54	12.6

exon in 5' UTR	66.46	63.33

intron in 5' UTR (5' 200 bp)	30.36	26.49

intron in 5' UTR	2.35	2.2

intron in 5' UTR (3' 200 bp)	24.08	21.07

initial coding exon	39.32	35.4

initial intron in coding area (5' 200 bp)	24.16	21.77

initial intron in coding area	1.76	1.7

initial intron in coding area (3' 200 bp)	20.17	18.05

internal exon	7.15	14.87

internal intron (5' 200 bp)	1.81	3.25

internal intron	0.39	0.78

internal intron (3' 200 bp)	1.55	2.83

terminal coding exon	13.01	11.75

terminal intron in coding area (5' 200 bp)	3.51	3.22

terminal intron in coding area	0.64	0.62

terminal intron in coding area (3' 200 bp)	3.05	2.77

exon in 3' UTR	1.93	1.82

intron in 3' UTR (5' 200 bp)	3.99	3.26

intron in 3' UTR	0.61	2.25

intron in 3' UTR (3' 200 bp)	2.8	0.56

The total length of CGI overlapping with exons and introns in different gene regions normalized for the expectation estimated from overlapping of randomly sampled intervals.

### In all gene regions synonymous substitution rates of codons that contain CpG dinucleotides are lower in exons overlapping with CGIs than in exons not overlapping with CGIs

**Figure 2:**
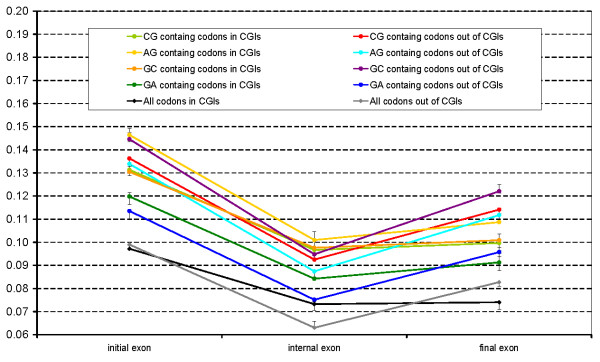
**d_N_**. Non-synonymous substitution rates calculated for various classes of codons overlapping and not overlapping with CGIs in different gene regions.

**Figure 3:**
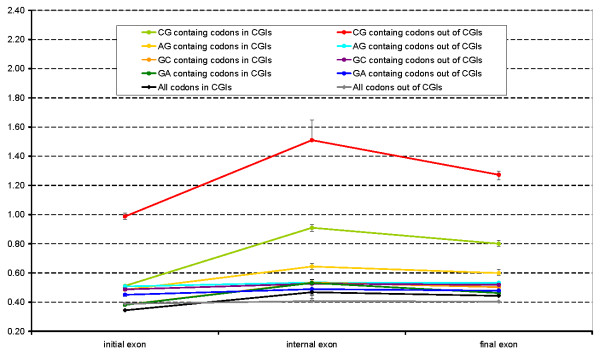
**d_S_**. Synonymous substitution rates calculated for various classes of codons overlapping and not overlapping with CGIs in different gene regions.

**Figure 4:**
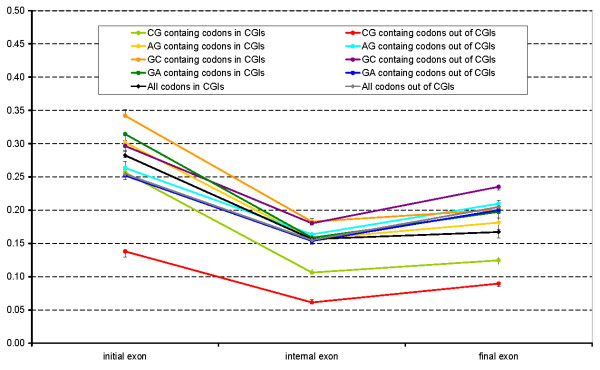
**d_N_/d_S_**. Synonymous to non-synonymous substitution rates ratio calculated for various classes of codons overlapping and not overlapping with CGIs in different gene regions.

The analysis above demonstrates CGI function is probably carried on at the level of nucleic acids. Therefore CGI presence can affect synonymous substitution rate for codons that overlap with CGIs. To test this, we compared synonymous (*d*_S_) and nonsynonymous (*d*_N_) substitution rates in human-mouse alignments for codons overlapping and non-overlapping with CGIs. Exons located in different parts of genes were considered separately. The substitution rates were calculated for all codons and separately for codons containing CpG, GpC, ApG and GpA dinucleotides. The results are presented in Table 2 and Figure 2, 3 and 4.

**Table 2:** d_N_, d_S _and d_N_/d_S_

Codon type	Initial exon			Internal exon			Final exon		
	***d***_**N**_	***d***_**S**_	***d***_**N**_***/d***_**S**_	***d***_**N**_	***d***_**S**_	***d***_**N**_***/d***_**S**_	***d***_**N**_	***d***_**S**_	***d***_**N**_***/d***_**S**_
CG containing codons in CGI	0.131	0.512	0.257	0.097	0.910	0.106	0.100	0.800	0.125

CG containing codons out of CGI	0.136	0.987	0.138	0.093	1.510	0.061	0.114	1.273	0.090

AG containing codons in CGI	0.146	0.485	0.302	0.101	0.644	0.157	0.109	0.599	0.181

AG containing codons out of CGI	0.134	0.508	0.264	0.087	0.535	0.164	0.112	0.533	0.210

GC containing codons in CGI	0.130	0.381	0.342	0.098	0.534	0.183	0.101	0.503	0.201

GC containing codons out of CGI	0.145	0.488	0.297	0.095	0.526	0.180	0.122	0.519	0.235

GA containing codons in CGI	0.120	0.381	0.314	0.084	0.531	0.159	0.091	0.463	0.197

GA containing codons out of CGI	0.114	0.450	0.252	0.075	0.489	0.154	0.096	0.479	0.200

All codons in CGIs	0.097	0.344	0.282	0.073	0.468	0.157	0.074	0.443	0.167

All codons out of CGIs	0.099	0.389	0.254	0.063	0.407	0.155	0.083	0.404	0.205

Rates were calculated separately for various gene elements and various classes of codons.

The nonsynonymous substitution rate for codons containing CpG dinucleotides was very similar to that for other codons and depended only weakly on the overlapping with CGIs (Figure 2). The main factor affecting rates of nonsynonymous substitutions is the codon location near one of the gene termini. Figure 2 shows "V"-shaped *d*_N _plots for all the codons outside of CGIs, which indicates that internal exons are less variable than both terminal exons. This effect may be related to the increased protein variability at the N and C termini. At the same time, codons overlapping with CGIs show almost equal *d*_N _for the internal and the final exons. Thus, proteins coded by genes having a CGI at their 3' end are generally more conserved at their C end.

In contrast, synonymous substitution rates calculated for codons containing CpG dinucleotides were different from those for other codons and dramatically depended on their overlapping with CGIs (Table 2 and Figure 3). Generally, for codons with CpG dinucleotides overlapping with CGI resulted in *d*_S _decrease approximately two-fold (Table 2 and Figure 3). For codons that did not contain CpG the effect of CGI on *d*_S_was much smaller. This effect did not depend on the gene region: a CGI overlapping with a 5', intragenic or 3' exon had a similar effect on *d*_S_, reducing the synonymous substitution rates of CpG containing codons by 49%, 40% and 37%, respectively.

Figure 4 shows the *d*_N_*/d*_S _ratio which reflects the selection pressure at the protein level [30]. For codons that do not contain CpG the *d*_N_*/d*_S _ratios are almost identical for codons that do overlap and don't overlap with CGIs. Thus, it appears that selection at the protein level for non CpG-containing codons inside or outside of CGIs is practically the same. For CpG-containing codons one can see that the *d*_N_*/d*_S _ratios calculated for codons overlapping and not overlapping with CGIs are substantially different, and both ratios are much lower (red and light green curve, Figure 4), which indicates a comparatively greater stabilizing selection at such codons at the protein level.

The observation that CpG containing codons have lower *d*_S _when they overlap with CGIs gives additional evidence that amino acid composition (e.g. abundance of arginine) cannot explain the abundance of CpG dinucleotides and the frequent overlap of CGIs and exons. Function of CGI indeed seems to be more related to DNA or RNA.

### Enrichment of CGIs with CAGE tags

In the previous sections we have demonstrated that in exons located far downstream from TSSs and overlapping with CGIs the synonymous substitution rate of CpG-containing codons is reduced. In addition, CGIs found far downstream from TSS often overlap with exons, but such CGIs are unlikely to be the misrecognized exons. Assuming that 5' related CGI are involved into transcription initiation [15,17] we have investigated if CGIs located in other genome regions also participate in transcription initiation. To test this suggestion we have studied association of computationally identified CGIs with transcription start sites as identified by CAGE tagging [25,26]. For our analysis we categorized CGIs into 4 non-overlapping classes: (1) 5' CGIs; (2) intragenic CGIs; (3) 3' CGIs; and (4) intergenic CGIs (see **Methods, CGI classes**). The number of CGI classes is smaller than the number of gene elements because the same CGI can often overlap with several gene elements, e.g. 5' UTR, the initial coding exon, the first intron, and sometimes other exons as well as downstream located introns.

CAGE tags exhibit a clear tendency to cluster within all classes of CGIs (Table 3). CGIs occupying about 0.7% of the entire genome contain more than 48% of all CAGE tags. About 70% of all CGIs contain at least one CAGE tag. In average 5', intragenic, 3', and intergenic CGIs contain respectively one CAGE tag per 20, 203, 172, and 86 base pairs as compared to the average genome CAGE frequency of 1 tag per 1,891 bp The frequency of CAGE tags in these CGIs is respectively 95-, 9-, 22-, and 11-fold greater than in the genome in average respectively with CGI class. A 5' CGI contains in average 44 CAGE tags; the number of CAGE tags in other classes of CGIs is 7- and 11-fold smaller.

**Table 3:** CAGE tags in different CGIs classes

CGI class	5prim	intragenic	3prim	intergenic	total
#CGIs	15686	3095	1808	6848	**27437**

Fraction of CGIs, %	57.17	11.28	6.59	24.96	**100**

Total length of CGIs	13853661	1483283	1124521	4482821	**20944286**

Average GC-content, %	66.66	65.8	66.53	66.39	**66.08**

#CGIs with CAGE-tags	13361	1327	1005	3509	**19202**

Total length of CGIs with CAGE-tags	12756213	784565	780040	2925761	**17246579**

Fraction of total length of CGIs with CAGE-tags in class, %	92.08	52.89	69.37	65.27	**82.35**

Total CAGE-tags in CGI class	698369	7300	6520	52377	**764566**

Fraction of CAGE-tags in CGI class, %	43.7	0.46	0.41	3.28	**47.85**

CGIs with at least one CAGE-tag, %	85.18	42.88	55.59	51.24	**69.99**

Average CAGE-tags per CGI	45	2	4	8	**28**

Average CAGE-tags per CAGE-containing CGI	52	6	6	15	**40**

Density of CAGE-tags in CGIs, bp^-1^	0.0504	0.0049	0.0058	0.0117	**0.0365**

Density of CAGE-tags in CAGE-containing CGIs, bp^-1^	0.0547	0.0093	0.0084	0.0179	**0.0443**

One CAGE-tag per #bp	20	203	172	86	**27**

Relative number of CGIs of different classes; CAGE tag representation and their frequency in different CGIs.

As it was already reported in [31] CAGE tags tend to form dense clusters in 5' CGIs. CGIs located elsewhere contain much less CAGE tags than 5' CGIs, but, interestingly, some intragenic, 3' or intergenic CGIs contain clusters of CAGE tags with the number and the density of CAGE tags comparable with those found in CAGE clusters in 5' CGIs. Additional file 1 contains intragenic and 3' CGIs that have greater than 40 CAGE tags per a CGI (which approximately corresponds to the average number of CAGE tags per 5' CGI). 3' CGIs usually contain more CAGE tags than intragenic CGIs. In some sense this agrees with the tendency of CGIs to overlap with the final coding exon rather than with internal exons.

### Not only 5'CGIs, but also 3', intragenic and intergenic CGIs are enriched with Sp1 binding sites

Authors of [15] reported that CGIs overlapping mouse promoters of housekeeping genes contained an increased number of binding sites for different transcription regulatory factors, in particular Sp1, ETS, and NRF-1. Since binding of Sp1 is well studied with experimental methods, we decided to assess Sp1 binding in CGIs of different localization relative to known genes. We used both bioinformatics methods of identification of Sp1 recognition motifs in DNA sequence as well as re-assessment of the published experimental data.

CGIs were scanned for presence of Sp1 factor binding sites using a positional weight matrix (PWM) constructed from experimental data from the TRANSFAC database. We selected a threshold that identified 90% of Sp1 binding sequences from our experimentally confirmed training set (see **Methods**). To evaluate the representation of Sp1 binding sites in CGIs, we calculated the *P*-value (see **Methods**) for each CGI, i.e. the probability of a random sequence of the same length and the same dinucleotide content to contain at least this number of Sp1 occurrences. This *P-value *was calculated with the help of the AhoPro program [32]. We compared results obtained for different CGI classes.

**Figure 5:**
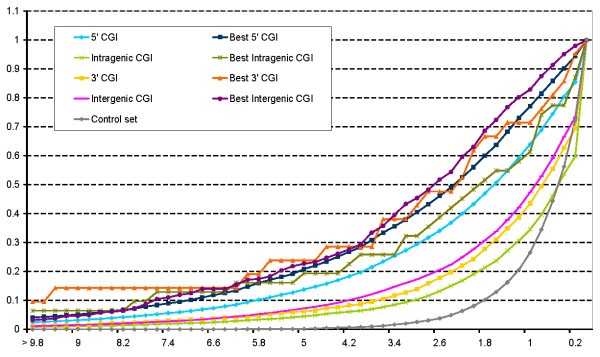
**Statistical significance of the relative occurrence of Sp1 binding sites within different CGI classes and GC-rich shuffled sequences**. X-axis: theoretical statistical significance (P-value); Y-axis: the overall fraction of sequences having a statistical significance less or equal than that at the X-axis. A higher statistical significance value reflects more Sp1 sites scoring above the PWM threshold within the selected CGI. CGI classes and GC-rich shuffled sequences are defined in **Methods**.

Figure 5 shows that for any PWM threshold, there are more Sp1 binding sites found in all types of CGIs including all non 5' CGI groups than in GC-rich control set. Although 5' CGIs contain more Sp1 binding sites than any CGIs, highly significant Sp1 hits (Figure 5, left) are represented to a similar degree in 3' and intergenic CGIs. Intragenic CGIs contain less Sp1 sites. For Sp1 sites of an intermediate quality, intergenic CGIs contain substantially more Sp1 binding sites than CGIs of any other class except for 5' CGIs.

It is noteworthy that CGIs containing more than 40 CAGE tags contain a much more high scoring Sp1 recognition motifs than CGIs without evidence of high transcription activity (Figure 5), independently from their localization in relation to genes. Surprisingly, the greatest overrepresentation of high-scoring Sp1 recognition motifs sites is characteristic for 3' CGIs with more than 40 CAGE tags, but not for 5' CGI enriched with CAGE.

### ChIP-chip data indicate that Sp1 factors tend to bind within CGIs

For further validation of Sp1 protein binding within CGIs, data on Sp1 transcription factor binding sites, experimentally assessed with ChIP-chip technology and published in [33] were analyzed. Cawley et al. detected frequent Sp1 binding sites far from 5' regions of any gene. We used their data to justify that Sp1 protein binds preferably within CpG islands regardless of their location in relation to genes.

Sp1 binding regions published in [33] are usually longer than 1 kB, which is significantly longer than many CGIs, especially those located far from TSS of genes. The authors of [33] used an extensive filtration procedure, which can lead to a high false negative rate, to limit their results to binding sites frequently occupied with Sp1. Therefore, the raw data were re-analyzed to allow comparison between ChIP signals within CGIs and those in other DNA segments. Additionally ChIP signals within CGIs located in different gene segments were examined.

**Figure 6:**
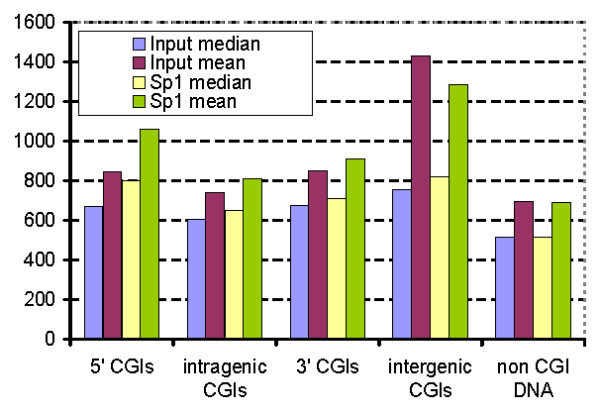
**ChIP-chip assessment of Sp1 binding in CGIs in different genome segments**. Mean and median intensities for Sp1 and input DNA signal for PM tags located in CGIs from different genome segments.

Figure 6 shows that signals of probes located within all types of CGIs are greater for Sp1 antibody treated samples than for the corresponding signals of control (the untreated input) samples. In contrast it is not possible to observe such a difference in non-CGI DNA. One can see that the control (untreated input) and the Sp1 antibody treated sample signals measured at tags overlapping with different CGI classes correlate, which is probably related to the increase in hybridization specificity with G and C content [34]. The distribution is highly skewed so the average in all cases is higher than the median. However, with one exception of intergenic CGIs, both the mean and the median of Affimetrix perfect match probe (PM) value distributions for Sp1 antibody treated samples are greater than the values of corresponding characteristics for control samples.

**Figure 7:**
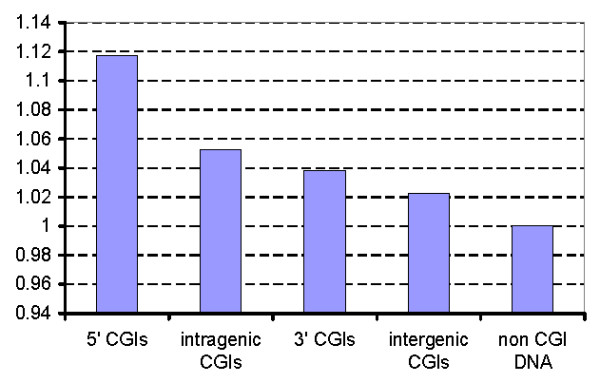
**ChIP-chip S/N ratio for Sp1 binding in CGIs in different genome segments**. Input/Sp1 signal ratio for PM tags located in CGIs from different genome segments.

Figure 7 shows the median of the signal ratios for the treatment and the control calculated for each tag. This value is presented for different CGI classes as well as for non-CGI DNA. All ratios are almost equal to one. As one can see from Figure 7 the binding signal of Sp1 is the greatest in CGIs located near 5' gene region; it is lesser in intergenic and 3' CGIs region and is missing in non-CGI DNA.

Since the difference between medians of hybridization signals for the input and the treated samples was in all cases very small we tested whether this difference was statistically significant using Wilcoxon-Mann-Whitney test statistics. Table 4 shows the P-values of the Wilcoxon-Mann-Whitney test statistics calculated for the input and the treated samples for different classes of CGIs. The test indicates that for all classes of CGIs the distribution of signal values from the Sp1 antibody treaded samples differ significantly (alpha = 5%) from the distribution of signal values of the corresponding control samples. In contrast the difference in non-CGI DNA is not significant.

**Table 4:** Statistical significance of Sp1 signal

Type of the region				
5' CGIs	Intragenic CGIs	3' CGIs	Intergenic CGIs	Non-CGI DNA
2.20E-16	4.12E-06	0.03978	0.0005911	0.08426

P-values of the Wilcoxon-Mann-Whitney tests performed on signal values from the Sp1 antibody treated sample and the corresponding control DNA sample within different CGI classes.

We also compared Sp1/input ratios between different classes of CGIs using Wilcoxon-Mann-Whitney test statistics. Table 5 shows that signal ratios from tags overlapping with CGIs of all types are significantly different from those in non-CGI DNA.

**Table 5:** Statistical significance of Sp1/input ratios

Type of the region				
5' CGIs	Intragenic CGIs	3' CGIs	Intergenic CGIs	Non-CGI DNA
X	<2.2E-16	2.75E-06	2.08E-05	<2.2E-16

X	X	1.44E-05	<2.2E-16	<2.2E-16

X	X	X	3.11E-10	<2.2E-16

X	X	X	X	<2.2E-16

The comparison of of Sp1/input ratios between different types of CGIs using Wilcoxon-Mann-Whitney test statistics.

### Non-5' CGIs with multiple CAGE tags are often associated with transcription starts sites of long RNAs for which no encoded proteins are known

We have explored if there are known transcripts associated with non-5'-CGIs enriched with CAGE tags. Table 6 demonstrates that 14 of 22 3' CGIs containing more then 40 CAGE tags are associated with a start of at least one potential coding gene from NCBI Reference Sequences (RefSeq). The corresponding value for intergenic CGIs is only 2 from 30. Other 8 of 22 3' CGIs and 28 of 30 intergenic CGIs also overlap with starts of long transcripts but without any evidence of a coded protein or at least a long ORF. To be exact, 5 3' CGIs and 18 intergenic CGIs contain start sites of mRNAs recorded in GeneBank. It should be mentioned that not all mRNAs in GeneBank are confirmed to code any protein; sometimes such RNAs only demonstrate mRNA properties, like having cap, polyA-tail or splicing. Therefore the mRNA database from GeneBank is likely to contain a fraction of long potentially non-coding RNAs. The remaining 3 3'CGIs and 10 intergenic CGIs contain start sites of spliced or unspliced ESTs. For CGIs containing from 20 to 40 CAGE tags the situation changes dramatically. In this case 29 of 41 3' CGIs contain starts of known long RNAs with no demonstrated protein-coding activity, whereas only 14 3'CGIs contain starts of protein-coding genes maintained in the RefSeq database. From all intragenic CGIs with 20-40 CAGE tags only 1 contains a start of a protein-coding gene and other 43 contain starts of mRNAs (or mRNA-like RNA) and ESTs. Thus, a substantial fraction of CAGE-enriched non 5'CGIs contains TSSs of long RNA showing no evidence of any encoded protein; this is especially true for CGIs with 20-40 CAGE tags.

**Table 6:** Transcript starts in non 5' CGI

Number of CGIs with starts of long transcripts					
**Type of CGIs**	**Unverified RefSeq genes**	**Gene Bank mRNA and mRNA-like RNA**	**Spliced and unspliced ESTs**	**No transcripts found**	**Total**

**CGIs with more that 40 CAGE-tags per CGI**					

3' CGI	14	5	3	0	22

Intragenic CGI	2	18	10	0	30

**CGIs with 20-40 CAGE-tags per CGI**					

3' CGI	12	14	11	4	41

intragenic CGI	1	19	24	0	44

Number of transcript starts in intragenic and 3' CGIs having more than 40 CAGE tags per a CGI.

The total number of 3' and intragenic CGI with more then 40 CAGE tags is rather small: 22 and 30 respectively. Decreasing the threshold for CAGE tags per CGI to 20-40 leads to 41 3' CGI and 44 intragenic CGIs. However, the number of highly CAGE-enriched non 5' CGIs is not large enough to render a convincing statistical significance value.

## Discussion

In this study we tried to systematically assess properties of CpG islands that are found far from transcription start sites of protein coding genes. About 43% of all CGIs belong to this class. Our study of CGIs which overlap with exons demonstrates that stabilizing selection protects CpG pairs located in CGIs from substitutions which do not affect the encoded amino acid sequence. We observed that many CGIs that are found far from TSS overlap with CAGE tags and thus participate in transcription; furthermore, highly CAGE-enriched CGIs are bound by transcription regulatory factor Sp1 with remarkably high significance. Although function of CGIs is still disputed, there is growing evidence that CGIs located near gene starts participate in transcription regulation [15,17,35]. Our finding allowed us to suggest that many CGIs that are found far from the start of any known protein coding gene are also participate in transcription.

As we have demonstrated, many such CGIs often overlap with exons, particularly the terminal gene exons. Many CGIs are located within a gene but far downstream from TSS (see Table 3). The aggregated number of genes with CGIs near their 3' end is estimated at 5 - 10%. Interestingly, it was observed recently [36] that some genes in human T-cells have an uncommon methylation pattern with a decreased methylation level observed near both gene termini.

We have detected many intergenic CGIs (see Table 3). It is known that UCSC browser table *Knowngenes *contains only highly verified genes, and excludes some genes with low justification scores. Based on our analysis, some CGIs considered in this study as intergenic may be related to these yet unverified genes.

**Figure 8:**
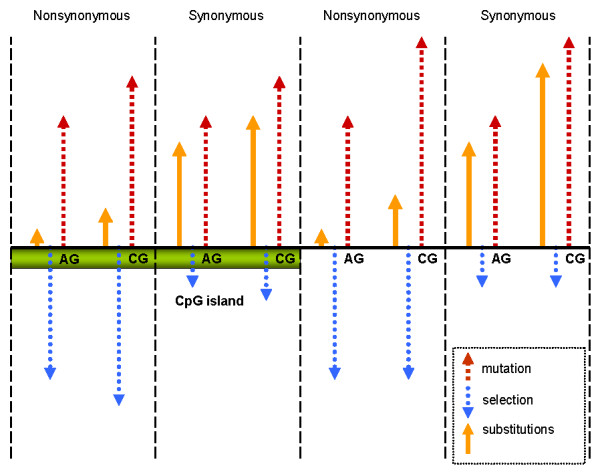
**Interaction between mutation process and selection pressure in exons overlapping and non-overlapping with CGIs**. In coding exons the substitution rate at synonymous sites is approximately 10-fold greater than at nonsynonymous sites. The ^m^CpG → TG transition rate is about 10-fold greater than AG -> GG transition rate. CpG islands protect CpG dinucleotides from methylation, decreasing the transition rate from CG to TG. CpG dinucleotides in CGIs may be under stronger selection than CpG dinucleotides not overlapping within CGIs.

CGIs located far downstream from TSS protect synonymous codon positions from substitutions very similar as do CGIs located near gene starts. A CGI is thought to reduce the CpG mutation rate by protecting DNA from methylation. On the other hand, CGIs probably contain many binding sites for transcription factors that overlap CpG dinucleotides [15]. Such binding can also increase conservation of CpG dinucleotides by applying stabilizing selection at nucleotide level that preserves functional binding sites. The decreased mutation rate and the purifying selective pressure would contribute to reduction of the substitution rate in CpG dinucleotides within CGIs (see Figure 8). This probably explains why the synonymous substitution rate in CpG containing codons in exon segments overlapping with CGIs becomes lower than that in exons not overlapping with CGIs. This effect is observed in all gene regions, 5' as well as 3' or intragenic, which supports the functional role of CGIs located in regions other than gene 5'.

The selection directed to maintain CGI sequence properties is not strong enough to overcome a strong selection at protein level applied to non-synonymous substitutions. The non-synonymous substitution rate differs only weakly for codons overlapping and non-overlapping with CGIs. However, the *d*_N_*/d*_S _ratio for CpG containing codons not overlapping with CGIs is much smaller than for codons overlapping with CGIs. This fact indicates that in this case selection at the protein level needs to be stronger to counterbalance the higher mutation rate.

The evolutionary distance between human and mouse is rather large, with the approximate sequence divergence for these species close to 0.5 substitutions for a neutrally evolving site [37]. This value agrees very well with the *d*_S _values observed for codons that do not contain CpG. However, *d*_S _values for CpG containing codons are about twice as large as those for other codons. This is much less than the approximate ten-fold increase of mutation rate [38]. The possible explanation may come from the effect suggested by Kondrashov et. al. [39]. The idea is that hypermutable CpG dinucleotides [40] at neutral and pseudoneutral sites are likely to be destroyed by mutations and unlikely to be found in the alignment of human and mouse [41-43]. Those CpG dinucleotides that remain aligned in human and mouse genomes are likely to be stabilized by selection pressure of a different nature. Thus, even CpG dinucleotides that do not overlap with CpG islands at synonymous positions may be stabilized with some selection of yet unknown type. Interestingly, Bock et al [44] who specifically identified CGIs related to chromatin epigenetic state observed that about 90% of such islands overlapped with highly conserved DNA elements, including at least 20% of CGIs that did not overlap with TSS.

Although CGI overlap with disproportionally large number of CAGE tags (about a half of total CAGE tags are found within CGIs) many intragenic, intergenic, and gene terminal CGIs overlap with a small number of CAGE tags or with no CAGE tags at all. However, we believe, that FANTOM database can have some functional transcription starts missing.

**Figure 9:**
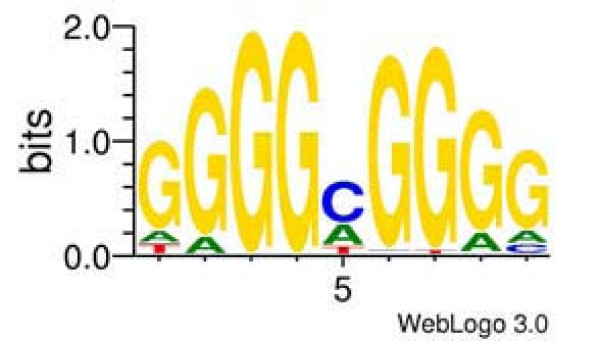
**Sequence logo for identified Sp1 site built using WebLogo **[59].

First, CAGE tags were mapped at the repeat-masked human genome, thus excluding so-called "GC-rich low-complexity regions" and simple repeats such as (CCCCG)_n_. Many CGIs contain such low complexity regions, and CAGE tags in these regions were excluded from our analysis. It is important to note that even a simple repeat such as (CCCCG)_n _can probably operate as functional Sp1 site (see Figure 9), and thus may play a role in CGI functioning. It is noteworthy that many computationally identified CGIs overlap with Alu repeats [18], therefore we did not filter out such CGIs, considering that they would only reduce the effect but not create an artifact.

Second, CAGE tags found in FANTOM database are obtained only in a number of tissues. CGIs located in 5' gene regions are usually found at starts of broadly expressed housekeeping genes [15]. Transcripts from TSS tagged in 3' regions of known genes could be tissue specific. Since the number of tissues studied is limited, the number of tagged TSS should be less than that in 5' gene regions.

The suggested role of non-5' CGIs in transcription initiation agrees with the excessive Sp1 binding in CGIs. The observation that Sp1 binding sites are often present in CGIs is not new [10]. It is noteworthy that CGIs enriched with CAGE tags contain more high-scoring Sp1 recognition motifs (Figure 5). Abundant Sp1 binding in gene 3' or intergenic regions was observed in genome-wide site location experiments [33], which reported that 36% of the clusters of Sp1, Myc and p53 binding sites lie within or immediately 3' to well-characterized genes. Authors of [33] assumed that in these cases, non-coding RNA transcription may be initiated.

Recent studies of the mouse genome [31] demonstrated that a large number of ncRNA are initiated in the 3' regions of the genes, with specific enrichment at the 3' terminus of the final exon. There are published reports which show that sometimes long ncRNA are synthesized to open large chromatin segments for subsequent transcription initiation [45,46]. On the other hand a substantial number of these ncRNAs are complementary to known genes as anti-sense strands, which has led to the suggestion of an additional mechanism of gene silencing by natural antisense interference [47]. The authors of [47] also observed that sense-antisense pairing was almost universally associated with candidate imprinted loci. As genetic imprinting is frequently associated with an altered methylation status of CpG islands [48,49], CGIs located in 3' gene regions [23,47] or intragenic CGIs [21,22] may play an important role in this process by regulation of gene expression via inducing antisense-based gene interference.

## Conclusions

Abundant non-coding transcripts discovered recently in all parts of a genome allow suggesting that there should be regulatory regions associated with transcription initiation of such RNA types. This agrees with a large number of CGIs not associated with transcription start site of any known protein coding gene. Here we demonstrate that many of such CGIs appear to be related to transcription initiation and at least some of them contain CpG pairs stabilized by natural selection. Expression of RNA controlled by promoters overlapping with these CGIs seems to be regulated by the same transcription factors as expression of protein coding genes, therefore these RNA molecules seem to be involved into the regulatory cascades and cellular processes possibly as non-coding RNA of some function. Additional studies, both experimental and *in silico *are needed to verify this hypothesis.

## Methods

We used a MySQL database and a set of Perl and Ruby scripts as analysis tools. The source code of the scripts, test sequence sets, and lists of genes under putative regulation by non-5' CGIs can be found at http://line.imb.ac.ru/CGI/

### DNA Sequence and Annotations

The sequence of the human genome (hg17) and the *Knowngene *Table were downloaded from http://genome.ucsc.edu/. Redundant copies of genes and multiple copies with the same name but different locations were removed. Genes with less then 3 exons were also excluded. The resulting set amounted to 35,915 entries, derived from an initial 39,368 entries in the *Knowngenes *Table. Table *Cpgislandext *(UCSC) was used as the set of CpG islands. We have excluded CGIs with 'random' chromosome location, retaining 27,437 out of 27,801 computationally annotated CGIs. All *Knowngenes *genes were taken into account when testing if there were any protein coding gene TSS near intergenic or 3' located CGIs. Tables *RefSeq *genes, *Human mRNA*, *Spliced EST *and *Human EST *(USCS) were used to find starts of potentially protein coding and noncoding genes.

### Gene elements definitions

We compiled 11 sets of gene fragments, defined as follows: (1) **5'-flank regions **began 3 kb upstream from TSS and extended till first found TSS. Overlaps with any transcribed sequence were excluded. (2) **5' UTR-exon regions **contained non-translated 5' exons (or exon segments); overlaps with any translated sequences were excluded. (3) **5' UTR-intron regions **contained introns separating non-translated exons or the last non-translated and the initial translated exons; overlaps with any translated sequences were excluded. (4) **Initial coding exons **contained entire initial coding exons for all gene or exon parts; overlapping exons of different isoforms were merged. (5) **Initial introns **contained introns separating the 1^st ^and the 2^nd ^coding exons, overlaps with regions included into groups (1-4) were excluded. (6) **Internal exons **contained all translated internal exons, excluding the initial and the final exons of any gene. (7) **Internal introns **contained all introns, but not the initial and the final introns and overlaps with any translated sequence. (8) **Final exons **contained the last translated exons or their parts. (9) **Final introns **contained the introns separating the final exons and the previous ones, excluding overlaps with any translated sequence. (10) **3' UTR exons **contained 3' non-translated exons or their parts, excluding overlaps with any translated sequence. (11) **3' UTR introns **contained introns separating the 3' non-translated exons or the 3' non-coding and the final coding exon, excluding overlaps with any translated sequence.

Since introns are usually much longer than exons, we also considered 200 bp intronic segments flanking the donor and the acceptor splice sites. The resulting regions, called "**intron terminal regions**" are comparable with exons in their length. We used 200 bp intron regions adjacent to exons as an additional control set to exclude the influence of the increased exon GC composition, which can be misinterpreted as CGI during computational identification. All genes elements are available in Additional file 2.

### CGI classes

We considered 4 different classes of CpG islands: (1) **5' CGIs **that overlapped with gene elements from groups 1-5 above; (2) **intragenic CGIs **that overlapped with gene elements from groups 6-7; (3) **3' CGIs **that overlapped with gene elements from groups 8-11 or with a region 3 kb downstream of any gene; and (4) **intergenic CGIs **that were located at least 3 Kb from any known gene upstream or downstream. All genes, including single and double-exon genes were taken into account in this case. If a CGI contained at least one bp of a 5' region of any gene it was considered as a 5' CGI regardless of how many additional regions it overlapped. If a CGI contained at least one bp of a 3' region of any gene, but not overlapped with its 5' region, it was considered as a 3' CGI. If a CGI contained at least one bp of a known gene, but not overlapped with its 5' or 3' region, it was considered as an intragenic CGI. A CGI was considered as intergenic if it did not belong to any of these classes. Additionally, we used a control set of CG-rich random sequences with the length and dinucleotide distribution estimated from each of CGIs containing more than 40 CAGE tags. The overall number and length of CGIs of different classes are given in Table 3. Since we took a special care to remove putative 5' CGIs from the other classes, the majority of all CGIs fells into 5' CGI class (Table 3). CGIs of all classes are available in Additional file 3.

### Evaluation of the statistical significance of overlaps between interval sets

Given two sets of non-overlapping genome intervals (e.g. CGIs and exons) we used 10,000 Monte-Carlo simulations to compute expected distribution of aggregated overlap length. All length distributions were computed independently on each chromosome. During simulations intervals of one set corresponded to genome coordinates of elements (e.g. CGIs) and the other set contained intervals with lengths corresponding to those of the second set of genome elements but located at random positions in the chromosome. Each run of simulations was repeated twice with a different "fixed" element set (see Figure 1). Program source (Ruby 1.8) and additional details are available in Additional file 4.

### Gene segments for substitution rates estimation

To estimate *d*_N _and *d*_S _we used the EDAS database [50], which contains 28,530 alignments of human and mouse genes. For genes with several isoforms, the longest isoform was taken. Genes with less than three coding exons were excluded. We also excluded genes which had less than 70% identity within protein alignment for any coding exon. The resulting dataset contained exons from 8,775 genes. Six groups of protein coding exon segments were defined: 5', internal, and 3' exons, overlapping and non-overlapping with CGIs. In each of these groups, we selected codons containing a CG dinucleotide; if a CG dinucleotide was split between two adjacent codons, both codons were taken. A similar procedure was performed for codons containing AG, GA, GC dinucleotides. Sequences from each group as well as codons containing CG, AG, GA, GC were concatenated.

### Estimation of substitution rates

The transitional to transversional substitution rate ratio (R), as well as the numbers of synonymous substitutions per synonymous site (*d*_S_) and nonsynonymous substitutions per nonsynonymous site (*d*_*N*_) were estimated by the Ina method [30]. Unlike maximum likelihood methods, this was effective for very long alignments (~3*10^6 ^bp), and was fast enough to allow bootstrap resampling. We used our own implementation of this method (developed in Perl). The 95% confidence intervals for evolutionary parameters were calculated using bootstrap percentiles [51]. 2000 bootstrap replications were used. Sequences of all groups and scripts used for estimation of substitution rates are available in Additional file 5.

### CAGE tags

The table of CAGE tags mapped on the RepeatMasked hg17 chromosome-build is available at http://gerg01.gsc.riken.jp/cage/download/hg17prmtr/cage.rep_tag.2005-01-16.chr_all_gff.tar.gz

This table contains 1,597,993 entries. We downloaded alignments of CAGE tag sequence with the genome region with a minimal identity of 0.88.

### Identification of Sp1 recognition motifs in DNA sequences

We used a positional weight matrix (PWM) [52,53] as a model. A PWM for Sp1 was constructed by aligning experimental data contained in the TRANSFAC [54] database. Sequences containing binding sites for human Sp1 (mostly footprints), were obtained from the TRANSFAC database (July 2007 release), mapped on the human genome, extracted with genome flanking sequences, and realigned using the SeSiMCMC Gibbs sampler [55] (see Additional file 6 for details). The most frequent sequence length between different SeSiMCMC runs was equal to 9 bp and we accepted that all motifs had this length. A PWM was constructed from the alignment obtained with SeSiMCMC using the formula described in [56]. The resulting alignment included 221 genome sequences (see motif logo in Figure 9).

### P-value calculation for Sp1 motif occurrences in sequences

To evaluate the P-value (i.e. to calculate the statistical significance of the observed number of Sp1 sites scoring higher than the fixed threshold in the test sequence) we used AhoPro [32]. For a test sequence containing *k *possibly overlapping PWM hits scoring higher than threshold *T*, the P-value was defined as the probability of observing no less than *k *such (possibly overlapping) hits in the random (i.i.d) sequence with the same nucleotide distribution and length as in the tested CGI.

### ChIP-chip data for Sp1 binding

Experiments in [33] were conducted on the Affymetrix GeneChip^® ^Human Tiling 1.0R Array Set. The results were downloaded from http://transcriptome.affymetrix.com/publication/tfbs/. Those chips contain unique 25 base-pair long sequence-tags for human chromosomes 21 and 22. The experiments for Sp1 were performed on two biological samples with three technical replicates for each chip. We used an modified version of TiMAT [57] to re-analyze the published cel-files. To allow comparison between different experiments the probes of each chip were median scaled to a signal value of 500. Additionally quantile-quantile [58] normalization was performed over all chips. The signal values from the two biological samples and the technical replicates were averaged to obtain one value for each probe. Signal values of biological probes of the Sp1 antibody treated and untreated control experiment were collected. As it is recommended in [57] mis-match probes (MM) were excluded and only perfect-match probes (PM) were considered for further investigation. Our aim was to compare the statistical distribution of PM values for tags located far from CGIs with PM values for tags overlapping different classes of CGIs as well as to compare signals for Sp1 antibody treated samples with those for untreated DNA.

## Abbreviations

CGI: CpG island; TSS: transcription start site; CAGE: cap analysis gene expression; ncRNA: non-coding RNA; EST: Expressed sequence tag; PM: Affimetrix perfect match probe; MM: Affimetrix mis-match probe

## Authors' contributions

YM participated in data preparation, carried out the data analysis, and contributed to results interpretation and the writing of the manuscript. MF contributed to data analyses and results interpretation. NO contributed to data preparation and analysis. DM contributed to data preparation for comparative genomics analysis. EE contributed to estimation of substitution rates. IK carried Monte Carlo simulations of statistical values and analysis of Sp1 binding sites. AH performed the analysis of ChIP-chip data. VM contributed to work coordination, results interpretation and writing of the manuscript. All authors read and approved the final manuscript.

## Supplementary Material

Additional file 1 **CAGE enriched non 5' CGI**. Tables contain lists of genes with CAGE-enriched CGIs in 3' and intragenic regions separately. Click here for file

Additional file 2 **Gene elements**. The archive contains row data used for statistical significance of gene elements and CGIs overlap. See Table 1 and Figure 1 in the manuscript. Click here for file

Additional file 3 **CGI classes**. The archive contains classes of CGIs used for calculation of CAGE tag frequency. Click here for file

Additional file 4 **Monte Carlo Simulations**. The archive contains Ruby scripts used to evaluate statistical significance of overlapping of gene segments and CGIs. Results of simulations with different "fixed" elements (see **Methods**) are also included. Click here for file

Additional file 5 **Comparative genomics**. The archive contains row data and Perl scripts to perform substitution rates calculation. Click here for file

Additional file 6 **Sp1**. This folder contains the data used for Sp1 binding sites prediction and detailed description of the procedure. Click here for file

## References

[B1] Bird AP DNA methylation and the frequency of CpG in animal DNA Nucleic Acids Res 1980 8 7 1499 1504 10.1093/nar/8.7.1499 6253938 PMC324012

[B2] Ahuja N Li Q Mohan AL Baylin SB Issa JP Aging and DNA methylation in colorectal mucosa and cancer Cancer Res 1998 58 23 5489 5494 9850084

[B3] Gardiner-Garden M Frommer M CpG islands in vertebrate genomes J Mol Biol 1987 196 2 261 282 10.1016/0022-2836(87)90689-9 3656447

[B4] Han L Su B Li WH Zhao Z CpG island density and its correlations with genomic features in mammalian genomes Genome Biol 2008 9 5 R79 10.1186/gb-2008-9-5-r79 18477403 PMC2441465

[B5] Lander ES Linton LM Birren B Nusbaum C Zody MC Baldwin J Devon K Dewar K Doyle M FitzHugh W Funke R Gage D Harris K Heaford A Howland J Kann L Lehoczky J LeVine R McEwan P McKernan K Meldrim J Mesirov JP Miranda C Morris W Naylor J Raymond C Rosetti M Santos R Sheridan A Sougnez C Initial sequencing and analysis of the human genome Nature 2001 409 6822 860 921 10.1038/35057062 11237011

[B6] Heisler LE Torti D Boutros PC Watson J Chan C Winegarden N Takahashi M Yau P Huang TH Farnham PJ Jurisica I Woodgett JR Bremner R Penn LZ Der SD CpG Island microarray probe sequences derived from a physical library are representative of CpG Islands annotated on the human genome Nucleic Acids Res 2005 33 9 2952 2961 10.1093/nar/gki582 15911630 PMC1137027

[B7] Cross SH Charlton JA Nan X Bird AP Purification of CpG islands using a methylated DNA binding column Nat Genet 1994 6 3 236 244 10.1038/ng0394-236 8012384

[B8] Eckhardt F Lewin J Cortese R Rakyan VK Attwood J Burger M Burton J Cox TV Davies R Down TA Haefliger C Horton R Howe K Jackson DK Kunde J Koenig C Liddle J Niblett D Otto T Pettett R Seemann S Thompson C West T Rogers J Olek A Berlin K Beck S DNA methylation profiling of human chromosomes 6, 20 and 22 Nat Genet 2006 38 12 1378 1385 10.1038/ng1909 17072317 PMC3082778

[B9] Yamada Y Watanabe H Miura F Soejima H Uchiyama M Iwasaka T Mukai T Sakaki Y Ito T A comprehensive analysis of allelic methylation status of CpG islands on human chromosome 21q Genome Res 2004 14 2 247 266 10.1101/gr.1351604 14762061 PMC327100

[B10] Macleod D Charlton J Mullins J Bird AP Sp1 sites in the mouse aprt gene promoter are required to prevent methylation of the CpG island Genes Dev 1994 8 19 2282 2292 10.1101/gad.8.19.2282 7958895

[B11] Weinmann AS Yan PS Oberley MJ Huang TH Farnham PJ Isolating human transcription factor targets by coupling chromatin immunoprecipitation and CpG island microarray analysis Genes Dev 2002 16 2 235 244 10.1101/gad.943102 11799066 PMC155318

[B12] Recillas-Targa F De La Rosa-Velazquez IA Soto-Reyes E Benitez-Bribiesca L Epigenetic boundaries of tumour suppressor gene promoters: the CTCF connection and its role in carcinogenesis J Cell Mol Med 2006 10 3 554 568 10.1111/j.1582-4934.2006.tb00420.x 16989720 PMC3933142

[B13] Briggs MR Kadonaga JT Bell SP Tjian R Purification and biochemical characterization of the promoter-specific transcription factor, Sp1 Science 1986 234 4772 47 52 10.1126/science.3529394 3529394

[B14] Holler M Westin G Jiricny J Schaffner W Sp1 transcription factor binds DNA and activates transcription even when the binding site is CpG methylated Genes Dev 1988 2 9 1127 1135 10.1101/gad.2.9.1127 3056778

[B15] Rozenberg JM Shlyakhtenko A Glass K Rishi V Myakishev MV FitzGerald PC Vinson C All and only CpG containing sequences are enriched in promoters abundantly bound by RNA polymerase II in multiple tissues BMC Genomics 2008 9 67 10.1186/1471-2164-9-67 18252004 PMC2267717

[B16] Larsen F Gundersen G Lopez R Prydz H CpG islands as gene markers in the human genome Genomics 1992 13 4 1095 1107 10.1016/0888-7543(92)90024-M 1505946

[B17] Saxonov S Berg P Brutlag DL A genome-wide analysis of CpG dinucleotides in the human genome distinguishes two distinct classes of promoters Proc Natl Acad Sci USA 2006 103 5 1412 1417 10.1073/pnas.0510310103 16432200 PMC1345710

[B18] Ponger L Duret L Mouchiroud D Determinants of CpG islands: expression in early embryo and isochore structure Genome Res 2001 11 11 1854 1860 11691850 10.1101/gr.174501 PMC311164

[B19] Graff JR Herman JG Myohanen S Baylin SB Vertino PM Mapping patterns of CpG island methylation in normal and neoplastic cells implicates both upstream and downstream regions in de novo methylation J Biol Chem 1997 272 35 22322 22329 10.1074/jbc.272.35.22322 9268383

[B20] Hackenberg M Previti C Luque-Escamilla PL Carpena P Martinez-Aroza J Oliver JL CpGcluster: a distance-based algorithm for CpG-island detection BMC Bioinformatics 2006 7 446 10.1186/1471-2105-7-446 17038168 PMC1617122

[B21] Ramser J Ahearn ME Lenski C Yariz KO Hellebrand H von Rhein M Clark RD Schmutzler RK Lichtner P Hoffman EP Meindl A Baumbach-Reardon L Rare missense and synonymous variants in UBE1 are associated with X-linked infantile spinal muscular atrophy Am J Hum Genet 2008 82 1 188 193 10.1016/j.ajhg.2007.09.009 18179898 PMC2253959

[B22] Smilinich NJ Day CD Fitzpatrick GV Caldwell GM Lossie AC Cooper PR Smallwood AC Joyce JA Schofield PN Reik W Nicholls RD Weksberg R Driscoll DJ Maher ER Shows TB Higgins MJ A maternally methylated CpG island in KvLQT1 is associated with an antisense paternal transcript and loss of imprinting in Beckwith-Wiedemann syndrome Proc Natl Acad Sci USA 1999 96 14 8064 8069 10.1073/pnas.96.14.8064 10393948 PMC22188

[B23] Takada S Tevendale M Baker J Georgiades P Campbell E Freeman T Johnson MH Paulsen M Ferguson-Smith AC Delta-like and gtl2 are reciprocally expressed, differentially methylated linked imprinted genes on mouse chromosome 12 Curr Biol 2000 10 18 1135 1138 10.1016/S0960-9822(00)00704-1 10996796

[B24] Shiraishi M Sekiguchi A Terry MJ Oates AJ Miyamoto Y Chuu YH Munakata M Sekiya T A comprehensive catalog of CpG islands methylated in human lung adenocarcinomas for the identification of tumor suppressor genes Oncogene 2002 21 23 3804 3813 10.1038/sj.onc.1205454 12032849

[B25] Carninci P Kasukawa T Katayama S Gough J Frith MC Maeda N Oyama R Ravasi T Lenhard B Wells C Kodzius R Shimokawa K Bajic VB Brenner SE Batalov S Forrest AR Zavolan M Davis MJ Wilming LG Aidinis V Allen JE Ambesi-Impiombato A Apweiler R Aturaliya RN Bailey TL Bansal M Baxter L Beisel KW Bersano T Bono H The transcriptional landscape of the mammalian genome Science 2005 309 5740 1559 1563 10.1126/science.1112014 16141072

[B26] Kawaji H Kasukawa T Fukuda S Katayama S Kai C Kawai J Carninci P Hayashizaki Y CAGE Basic/Analysis Databases: the CAGE resource for comprehensive promoter analysis Nucleic Acids Res 2006 34 Database D632 636 10.1093/nar/gkj034 16381948 PMC1347397

[B27] Takai D Jones PA Comprehensive analysis of CpG islands in human chromosomes 21 and 22 Proc Natl Acad Sci USA 2002 99 6 3740 3745 10.1073/pnas.052410099 11891299 PMC122594

[B28] Takai D Jones PA The CpG island searcher: a new WWW resource In Silico Biol 2003 3 3 235 240 12954087

[B29] Aissani B D'Onofrio G Mouchiroud D Gardiner K Gautier C Bernardi G The compositional properties of human genes J Mol Evol 1991 32 6 493 503 10.1007/BF02102651 1908020

[B30] Ina Y New methods for estimating the numbers of synonymous and nonsynonymous substitutions J Mol Evol 1995 40 2 190 226 10.1007/BF00167113 7699723

[B31] Carninci P Sandelin A Lenhard B Katayama S Shimokawa K Ponjavic J Semple CA Taylor MS Engstrom PG Frith MC Forrest AR Alkema WB Tan SL Plessy C Kodzius R Ravasi T Kasukawa T Fukuda S Kanamori-Katayama M Kitazume Y Kawaji H Kai C Nakamura M Konno H Nakano K Mottagui-Tabar S Arner P Chesi A Gustincich S Persichetti F Genome-wide analysis of mammalian promoter architecture and evolution Nat Genet 2006 38 6 626 635 10.1038/ng1789 16645617

[B32] Boeva V Clement J Regnier M Roytberg MA Makeev VJ Exact p-value calculation for heterotypic clusters of regulatory motifs and its application in computational annotation of cis-regulatory modules Algorithms Mol Biol 2007 2 13 10.1186/1748-7188-2-13 17927813 PMC2174486

[B33] Cawley S Bekiranov S Ng HH Kapranov P Sekinger EA Kampa D Piccolboni A Sementchenko V Cheng J Williams AJ Wheeler R Wong B Drenkow J Yamanaka M Patel S Brubaker S Tammana H Helt G Struhl K Gingeras TR Unbiased mapping of transcription factor binding sites along human chromosomes 21 and 22 points to widespread regulation of noncoding RNAs Cell 2004 116 4 499 509 10.1016/S0092-8674(04)00127-8 14980218

[B34] Koltai H Weingarten-Baror C Specificity of DNA microarray hybridization: characterization, effectors and approaches for data correction Nucleic Acids Res 2008 36 7 2395 2405 10.1093/nar/gkn087 18299281 PMC2367720

[B35] Chung JH Bell AC Felsenfeld G Characterization of the chicken beta-globin insulator Proc Natl Acad Sci USA 1997 94 2 575 580 10.1073/pnas.94.2.575 9012826 PMC19555

[B36] Choi JK Bae JB Lyu J Kim TY Kim YJ Nucleosome deposition and DNA methylation at coding region boundaries Genome Biol 2009 10 9 R89 10.1186/gb-2009-10-9-r89 19723310 PMC2768978

[B37] Kumar S Subramanian S Mutation rates in mammalian genomes Proc Natl Acad Sci USA 2002 99 2 803 808 10.1073/pnas.022629899 11792858 PMC117386

[B38] Fujita N Shimotake N Ohki I Chiba T Saya H Shirakawa M Nakao M Mechanism of transcriptional regulation by methyl-CpG binding protein MBD1 Mol Cell Biol 2000 20 14 5107 5118 10.1128/MCB.20.14.5107-5118.2000 10866667 PMC85960

[B39] Kondrashov FA Ogurtsov AY Kondrashov AS Selection in favor of nucleotides G and C diversifies evolution rates and levels of polymorphism at mammalian synonymous sites J Theor Biol 2006 240 4 616 626 10.1016/j.jtbi.2005.10.020 16343547

[B40] Arndt PF Burge CB Hwa T DNA sequence evolution with neighbor-dependent mutation J Comput Biol 2003 10 3-4 313 322 10.1089/10665270360688039 12935330

[B41] Subramanian S Kumar S Neutral substitutions occur at a faster rate in exons than in noncoding DNA in primate genomes Genome Res 2003 13 5 838 844 10.1101/gr.1152803 12727904 PMC430942

[B42] Subramanian S Kumar S Evolutionary anatomies of positions and types of disease-associated and neutral amino acid mutations in the human genome BMC Genomics 2006 7 306 10.1186/1471-2164-7-306 17144929 PMC1702542

[B43] Subramanian S Kumar S Higher intensity of purifying selection on >90% of the human genes revealed by the intrinsic replacement mutation rates Mol Biol Evol 2006 23 12 2283 2287 10.1093/molbev/msl123 16982819 PMC3072915

[B44] Bock C Walter J Paulsen M Lengauer T CpG island mapping by epigenome prediction PLoS Comput Biol 2007 3 6 e110 10.1371/journal.pcbi.0030110 17559301 PMC1892605

[B45] Ho Y Elefant F Liebhaber SA Cooke NE Locus control region transcription plays an active role in long-range gene activation Mol Cell 2006 23 3 365 375 10.1016/j.molcel.2006.05.041 16885026

[B46] Gribnau J Diderich K Pruzina S Calzolari R Fraser P Intergenic transcription and developmental remodeling of chromatin subdomains in the human beta-globin locus Mol Cell 2000 5 2 377 386 10.1016/S1097-2765(00)80432-3 10882078

[B47] Katayama S Tomaru Y Kasukawa T Waki K Nakanishi M Nakamura M Nishida H Yap CC Suzuki M Kawai J Suzuki H Carninci P Hayashizaki Y Wells C Frith M Ravasi T Pang KC Hallinan J Mattick J Hume DA Lipovich L Batalov S Engstrom PG Mizuno Y Faghihi MA Sandelin A Chalk AM Mottagui-Tabar S Liang Z Lenhard B Antisense transcription in the mammalian transcriptome Science 2005 309 5740 1564 1566 10.1126/science.1112009 16141073

[B48] Teodoridis JM Strathdee G Brown R Epigenetic silencing mediated by CpG island methylation: potential as a therapeutic target and as a biomarker Drug Resist Updat 2004 7 4-5 267 278 10.1016/j.drup.2004.06.005 15533764

[B49] Hirota T Ieiri I Takane H Maegawa S Hosokawa M Kobayashi K Chiba K Nanba E Oshimura M Sato T Higuchi S Otsubo K Allelic expression imbalance of the human CYP3A4 gene and individual phenotypic status Hum Mol Genet 2004 13 23 2959 2969 10.1093/hmg/ddh313 15459178

[B50] Nurtdinov RN Neverov AD Mal'ko DB Kosmodem'ianskii IA Ermakova EO Ramenskii VE Mironov AA Gel'fand MS [EDAS, databases of alternatively spliced human genes] Biofizika 2006 51 4 589 592 16909834

[B51] Efron B Tibshirani R An Introduction to the Bootstrap 1994 New York: Chapman & Hall/CRC

[B52] Berg OG von Hippel PH Selection of DNA binding sites by regulatory proteins. Statistical-mechanical theory and application to operators and promoters J Mol Biol 1987 193 4 723 750 10.1016/0022-2836(87)90354-8 3612791

[B53] Stormo GD Schneider TD Gold L Quantitative analysis of the relationship between nucleotide sequence and functional activity Nucleic Acids Res 1986 14 16 6661 6679 10.1093/nar/14.16.6661 3092188 PMC311672

[B54] Matys V Kel-Margoulis OV Fricke E Liebich I Land S Barre-Dirrie A Reuter I Chekmenev D Krull M Hornischer K Voss N Stegmaier P Lewicki-Potapov B Saxel H Kel AE Wingender E TRANSFAC and its module TRANSCompel: transcriptional gene regulation in eukaryotes Nucleic Acids Res 2006 34 Database D108 110 10.1093/nar/gkj143 16381825 PMC1347505

[B55] Favorov AV Gelfand MS Gerasimova AV Ravcheev DA Mironov AA Makeev VJ A Gibbs sampler for identification of symmetrically structured, spaced DNA motifs with improved estimation of the signal length Bioinformatics 2005 21 10 2240 2245 10.1093/bioinformatics/bti336 15728117

[B56] Lifanov AP Makeev VJ Nazina AG Papatsenko DA Homotypic regulatory clusters in Drosophila Genome Res 2003 13 4 579 588 10.1101/gr.668403 12670999 PMC430164

[B57] Biemar F Nix DA Piel J Peterson B Ronshaugen M Sementchenko V Bell I Manak JR Levine MS Comprehensive identification of Drosophila dorsal-ventral patterning genes using a whole-genome tiling array Proc Natl Acad Sci USA 2006 103 34 12763 12768 10.1073/pnas.0604484103 16908844 PMC1636694

[B58] Bolstad BM Irizarry RA Astrand M Speed TP A comparison of normalization methods for high density oligonucleotide array data based on variance and bias Bioinformatics 2003 19 2 185 193 10.1093/bioinformatics/19.2.185 12538238

[B59] Crooks GE Hon G Chandonia JM Brenner SE WebLogo: a sequence logo generator Genome Res 2004 14 6 1188 1190 10.1101/gr.849004 15173120 PMC419797

