# Supplementary figures and images for: Intergenic, gene terminal, and intragenic CpG islands in the human genome
Source: BMC Genomics. 2010 Jan 19;11:48. doi: 10.1186/1471-2164-11-48 (PMC2817693; doi:10.1186/1471-2164-11-48)

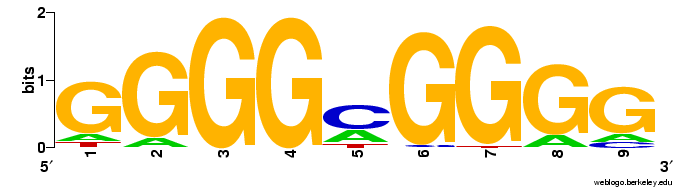

Supplement: Additional file 6 — Sp1. This folder contains the data used for Sp1 binding sites prediction and detailed description of the procedure. [file 1471-2164-11-48-S6.ZIP › SP1/sp1_site_extraction/9r.png]
